# Anticipatory Behavior and Enrichment: Insights into Assessing and Managing Harbor Seal (*Phoca vitulina richardii*) Pup Welfare in a Wildlife Rehabilitation Setting

**DOI:** 10.3390/ani15223237

**Published:** 2025-11-07

**Authors:** Karli R. Chudeau, Sophie Guarasci, Bethany Krebs, Cara Field, Jason V. Watters

**Affiliations:** 1 Animal Behavior Graduate Group, University of California, Davis, Davis, CA 95616, USA; 2 The Marine Mammal Center, Sausalito, CA 94965, USA; 3 Independent Researcher, San Francisco, CA 94312, USA

**Keywords:** animal welfare, animal behavior, cognitive enrichment, subjective states, harbor seals, pinnipeds

## Abstract

**Simple Summary:**

Caring for animals in wildlife rehabilitation requires not only meeting their physical needs but supporting their emotional well-being. However, it is difficult to measure how animals feel in this setting without using intensive methods. One promising way to do this is by observing “anticipatory behavior,” which is how animals act when they are expecting something positive within their environment, like a scheduled feed. This behavior can reveal how strongly animals expect or need rewarding experiences, and offers insight into their welfare. In our study, we worked with fourteen harbor seal pups during rehabilitation. Each day we provided the pups with different types of environmental enrichment, such as objects to explore (structural enrichment) or tasks that challenged their problem-solving abilities (cognitive enrichment). We then observed the pups’ behavior before feedings to see how the enrichment influenced their anticipation of food. Although both forms of enrichment were engaging, we found a trend suggesting pups given problem-solving tasks showed shorter periods of anticipatory behavior than seals given objects to explore. This finding suggests that cognitive enrichment may help improve emotional well-being of seals during rehabilitation. Using this approach, caretakers may better be able to support the welfare of marine mammals in their care.

**Abstract:**

The assessment of animal welfare in rehabilitation settings is a critical aspect of effective care, yet typical metrics often fail to fully capture rehabilitating animals’ emotional experiences in a non-invasive way. Anticipatory behavior has emerged as a promising animal welfare indicator, reflecting an animal’s perceived need for rewards based on available opportunities in their environment. By tracking anticipatory responses, caretakers can gain insight into an animal’s reward sensitivity and use this information to guide management interventions. This study investigated the effects of enrichment type on anticipatory behavior in fourteen, rehabilitating harbor seal pups (*Phoca vitulina richardii*). We provided pups with daily sessions of either structural or cognitive enrichment and recorded their behavioral responses. During scheduled feeding sessions, we identified behaviors that emerged as anticipatory, then measured the frequency and duration of anticipatory behavior prior to the feeds to assess how enrichment types influenced the seals’ reward sensitivity, and thus their welfare. While enrichment interaction did not directly modulate anticipatory behavior, we observed a trend suggesting that exposure to cognitive enrichment reduced anticipatory behavior duration compared to structural enrichment. These findings align with previous research in zoo settings, where cognitive enrichment has been linked to improved welfare through reduced anticipatory behavior, though this effect has not been explored in a wildlife rehabilitation context. This study highlights the value of anticipatory behavior as a practical welfare assessment tool in rehabilitation settings and underscores the potential for enrichment, particularly cognitive, to improve welfare in rehabilitating marine mammals.

## 1. Introduction

**Figure 1:**
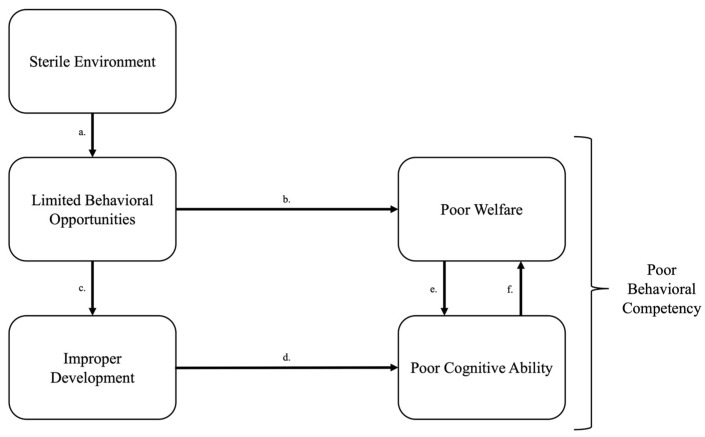
Cascading effects of a sterile environment on welfare. (a) A sterile environment reduces opportunities for animals to express a wide repertoire of behaviors and stunts fundamental “Needs” of information gathering, reward seeking, and exerting control [7]. (b) Inability to express intrinsically motivated behavioral needs leads to boredom, stress, and frustration [7]. (c) Limited behavioral opportunities prompt improper neural and behavioral development [9,12] which (d) leads to animals responding poorly to cognitive challenges or not responding at all, thus reducing behavioral competency [13]. (e) The lack of opportunity to develop appropriate behaviors increase boredom, stress, and frustration [14]. (f) Poor cognitive abilities and welfare turn into a feedback loop where poor cognitive ability increases stress and stress impairs learning, the combined deficiencies reducing behavioral competency’s ability to flexibly respond to environmental challenges or opportunities.

In the unique setting of wildlife rehabilitation, humans rescue and temporarily care for ill, injured, or displaced wild animals with the goal of reintroducing them to thrive in their native environment [1,2]. Wildlife rehabilitation is a complex, evolving process that unfolds into two distinct phases: the veterinary phase, which addresses immediate clinical health needs, and the reconditioning phase which on focuses on the animals’ physical and psychological preparation for release back into the wild [2]. While veterinary care is essential for physical recovery, welfare extends beyond physical health. Here, we define welfare as the quality of an individual animal’s subjective experience or emotional state [3,4,5]. Sterile environments or environments where an animal cannot exert control may stunt evolutionarily rooted motivations such as seeking information from the environment and responding to the environment in a way that will lead to reward acquisition (Figure 1) [6,7]. Furthermore, limited behavioral opportunities due to sterile environments are detrimental specifically to young animals who rely on dynamic, stimulating environments for proper brain and behavioral development (Figure 1) [8,9,10]. Animals destined for release into the wild require developmentally appropriate modifications to their managed care environments to prepare them to respond flexibly upon release [11]. Without considering the psychological and cognitive needs of the animal, an animal in peak physical condition may still struggle to maintain positive emotional states in a sterile rehabilitation environment and may not be prepared for survival in a wild, post-release environment. Thus, bringing an animal back to baseline health levels alone cannot be interpreted as evidence of a positive welfare state, nor sufficient to prepare rehabilitated animals to thrive upon release. This highlights the importance of supporting animal welfare during wildlife rehabilitation.

Wildlife rehabilitation facilities require increased sanitation and safety measures for animal health, and minimization of human interaction to prevent habituation that supports successful reintroduction into the wild environment, specifically for neonate and weanling animals prematurely separated from mothers that are more susceptible to habituation. The necessary constraints on environmental complexity in wildlife rehabilitation present welfare challenges, as barren, under-stimulating environments negatively impact subjective state of animals [15]. Environmental enrichment has emerged as a promising tool for wild animals in temporary care, offering animals opportunities for stimulation and behavioral expression [2,16,17]. In rehabilitation settings, the benefits of environmental enrichment may be even more pronounced for young animals who rely on dynamic, stimulating environments for proper brain and behavioral development [8,9,10]. Previous research in multiple animal care fields demonstrates that enrichment, in general, decreases abnormal behaviors, improves cognitive brain development, and promotes normal development and species-typical behaviors [9,18,19,20]. Enrichment research is now evolving to determine if there are certain types of enrichment, such as cognitive enrichment, that provide greater welfare benefits than other types [8,21,22]. Given the potential importance of the welfare of rehabilitated animals to their post-release success, understanding how to optimally apply environmental enrichment to support positive welfare for animals in rehabilitation is critical. However, making sure behavioral interventions, such as environmental enrichment, have the desired welfare impact must be a priority.

To ensure this welfare impact, pairing welfare assessments with applications of environmental enrichment can help identify which methods are the most effective. For the purposes of this study, the definition of welfare we subscribe to includes understanding the balance of pleasant and unpleasant emotional states [3,4,5]. Behavior offers a window into animals’ emotional states, as it is the first response to environmental change and shaped by past and present experiences [5]. Because behavior reflects both physical and psychological experiences, it can serve as a more direct indicator of welfare than physiological/health metrics alone. One method which uses behavior to assess animals’ affective states is cognitive bias testing, which assesses the animal’s emotional state through their response to ambiguous stimuli [23]. Evidence shows that decision-making behaviors of how to respond to ambiguous stimuli provide insight into an animal’s underlying emotional state [24]. Emotional states are foundational components of a proximate mechanism that adaptively guides behavior and decision-making to increase fitness. This link between emotional states and decision-making has guided cognitive testing to measure conditioned behavioral responses that infer an animal’s emotional state [13,25]. Cognitive bias testing is unfortunately incompatible with the goals of rehabilitation, as it requires extensive positive reinforcement training by caretakers which would negate the efforts to avoid habituation to humans in a rehabilitation context. A promising alternative measure of welfare which provides a graded assessment of an animal’s emotional state, and requires little human intervention is the measurement of anticipatory behavior [26,27].

Anticipatory behavior is a suite of goal-directed behaviors that animals exhibit prior to acquisition of a desired resource which occur in response to timing or environmental cues [26,27,28]. Anticipatory behavior has been well-documented across species in a variety of managed care settings [29,30,31,32,33]. Developed through learned associations with environmental cues (which may or may not be apparent to caretakers), anticipatory behavior predicts the acquisition of a desired resource such as housing [32], food [31,34,35], enrichment [28,29,36,37], social interactions [30,38], or positive reinforcement training [12,39]. Anticipatory behavior reflects the perception of the animal’s own reward sensitivity by combining an animal’s internal state with past experiences and placing a reward value on the upcoming resource that the animal will access [4,37]. Alternatively stated, an animal’s welfare state is captured through anticipatory behavior by revealing how much the animal believes they “need” the upcoming reward based on their own perception of available rewarding opportunities in the environment with the intensity of anticipatory behavior expression serving as a graded indicator of welfare rather than a one-dimensional positive or negative welfare indicator [26,27].

Our study aimed to investigate whether anticipatory behavior can be used as a welfare measure in a wildlife rehabilitation setting and whether different types of enrichment uniquely modulate anticipatory behavior in rehabilitating harbor seal pups. Chudeau and colleagues (2019) found that enrichment in a rehabilitation setting is an effective tool to improve harbor seal (*Phoca vitulina richardii*) pups’ welfare and promote foraging behavior, using a standard control versus treatment design [16]. However, we do not yet know how different types of enrichment, such as cognitive enrichment, influence welfare in this setting.

The goal of this research is to test the efficacy of anticipatory behavior as a welfare metric by investigating how different types of enrichment and degrees of engagement with enrichment modulate the welfare of fourteen harbor seal pups while in rehabilitation, as expressed by their anticipatory behavior. By exploring how this age-class and species express anticipation in this setting, we can better refine welfare assessments and test what management strategies, such as environmental enrichment, improve welfare and modulate behavior while in human care. Given that enrichment is well-established as a management tool to improve welfare, if anticipatory behavior decreases with increased enrichment interaction, this would support its utility as a sensitive welfare indicator in this unique setting.

We provided seals with enrichment from two different categories, structural and cognitive. Our first hypothesis (H1) is that enrichment interaction will modulate anticipatory behavior, such that seals with higher enrichment interaction will be more likely to exhibit lower anticipatory behavior rates prior to feeding sessions. Our second hypothesis (H2) is that enrichment type further modulates anticipatory behavior, such that increased exposure to cognitive enrichment will result in lower rates of anticipatory behavior compared to increased exposure to structural enrichment.

## 2. Methods

### 2.1. Subjects

In the springs of 2021 and 2022, trained and authorized personnel rescued Eastern Pacific harbor seal (*Phoca vitulina richardii*) pups that stranded (i.e., required intervention due to injury, illness, or premature maternal separation) on beaches between San Luis Obispo County and Mendocino County. Personnel transported seals to The Marine Mammal Center (TMMC), a wildlife rehabilitation hospital based in the Marin Headlands of Sausalito, California. Veterinary staff completed a full examination of pups upon admission to establish age, sex, and a primary diagnosis for stranding to determine the course of rehabilitative care. Neonatal pups received subcutaneous fluids as needed and husbandry crews fed them a formula of high-protein milk powder (Zoologic^®^ Milk Matrix 30/52; PetAg, Inc., Hampshire, IL, USA) containing low levels of lactose and fortified with vitamins, minerals, and salmon oil via tube feeding three to four times daily. Once seal pups’ teeth buds erupted and their health stabilized, veterinary staff gradually integrated whole, thawed fish into the seals’ daily diet and tube feedings discontinued once seals were reliably eating whole fish.

We housed seal pups with conspecifics in groups of up to eight individuals of similar developmental age (as determined by health status and a pup’s ability to process and consume fish) in one of four outdoor, concrete enclosures (5.2 × 3.7 m) containing an in-ground, 5488 L pool (3.0 × 2.4 m). All pools are part of a closed, non-heated recirculating water system with salinity maintained at 24–30 parts per thousand and water filtered and disinfected by ozone filtration with continuous water turnover rate of 30 min. We designated each enclosure as either a structural enrichment pool or a cognitive enrichment pool for the duration of the stranding season. Once pups were independently eating frozen/thawed herring (henceforth: free-feeding) proficiently and in stable health, staff transferred them to one of two larger, “pre-release” enclosures (6.1 × 4.6 m) containing an above-ground, 6912 L pool (2.4 × 2.4 m), where pups continued to gain weight in order to qualify for release. We also designated these as either structural or cognitive enrichment enclosures.

#### Subjects: Inclusion Criteria

Once deemed stable by veterinary staff, we randomly assigned pups to either the structural enrichment or cognitive enrichment condition, primarily depending on vacancies in those enclosures. As veterinary staff needed to move pups from one enclosure to the other for husbandry reasons, they took care to move pups that were in a certain condition into another enclosure with the equivalent condition (e.g., pups in structural enrichment enclosures at the beginning of rehabilitation moved to another structural enrichment enclosure for the duration of rehabilitation). While all seals admitted to TMMC received daily enrichment as part of their standard husbandry, to be included in this study we used similar criteria as Chudeau and colleagues (2019), where seals (a) had a malnutrition and/or maternal separation diagnosis (no trauma or clinical disease diagnoses), (b) had no health issues that required additional veterinary intervention after transfer into the enclosures, and (c) required no medication during care aside from a short course of antibiotics given upon admit to prevent umbilical infection. In addition to this inclusion criteria, prior to data analysis, we excluded seals who were exposed to our enrichment conditions for less than 21 days prior to release. Of 56 potential subjects across both seasons, we included 14 pups in our final sample. To identify each pup individually while in rehabilitation, we used small, flat 3D-printed hat-tags made of biodegradable material (Planetary Health Solutions) attached to the fur on their heads with unique ID numbers. We also used flipper tags that are required by federal rehabilitation standards and intended to be a long-term identifier to facilitate individual identification post-release as well as in rehabilitative care.

### 2.2. Procedure

#### 2.2.1. Enrichment Sessions

Animal care personnel (henceforth: crews) provided two enrichment sessions per day to every pool, each for 30 min, the timing of which varied between the hours of 7:00 and 22:00, independently from the feeding sessions. Enrichment sessions began when crews placed a randomly assigned enrichment device in the pool and ended when crews removed the device from the pool. We used Axis P3245-LVE cameras (Lund, Sweden) to record enrichment sessions and downloaded footage remotely using ExacqVision software (version 21.12.2.0) for later behavioral analysis.

#### 2.2.2. Enrichment Stimuli

**Figure 2:**
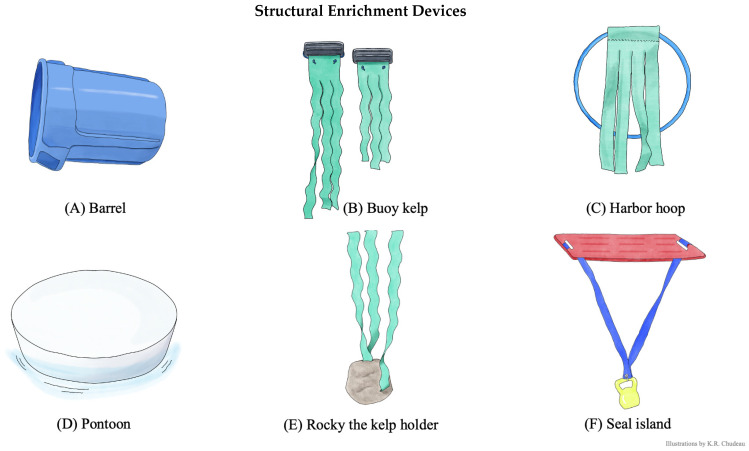
Structural enrichment devices. Structural enrichment device components and common behaviors observed. Illustrations by K.R. Chudeau. (**A**) Barrel. A 75.7 L plastic rubbish bin container with the 49.5 cm diameter bottom cut out. Seals swam through the barrel like a tunnel and would swim on top and push around. (**B**) Buoy kelp. Two vinyl boat fender buoys attached to untreated car wash “mitter curtains” (henceforth: artificial kelp). Seals pushed floats, pulled on and wrapped themselves in artificial kelp. (**C**) Harbor hoop. Weighted hula hoop with artificial kelp attached. Seals swam through, pushed around hoop, and pulled on artificial kelp. (**D**) Pontoon. 134.6 cm diameter × 13.9 cm high animal-safe, food-grade polyethylene plastic pill float from Wildlife Toybox. Seals swam underneath and attempt to haul out on the top surface. (**E**) Rocky the kelp holder. Artificial kelp secured to a 3.6 kg vinyl-coated kettle bell and weaved through holes created in a 45.7 cm diameter × 61 cm high, artificial resin rock. Seals pulled on and swam through artificial kelp and would push on the weighted rock. (**F**) Seal island. A 121.9 × 76.2 cm plastic herding board attached to a 3.6 kg vinyl-coated kettle bell using artificial kelp. Seals swam underneath and hauled out on the top surface.

**Figure 3:**
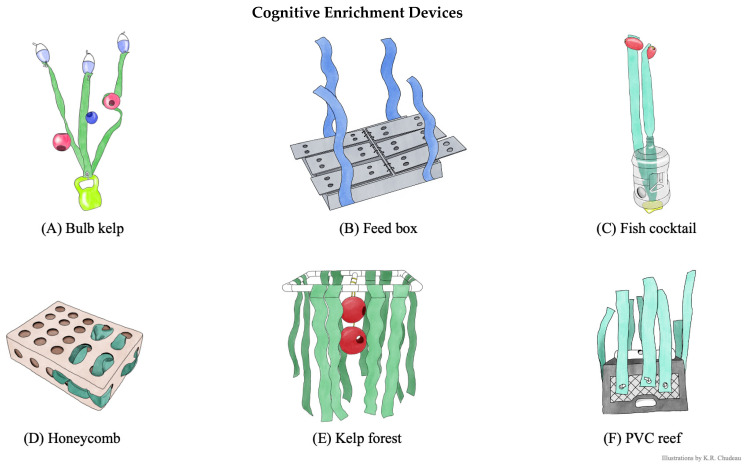
Cognitive enrichment devices. Cognitive enrichment device components and the problem-solving goals. Illustrations by K.R. Chudeau. (**A**) Bulb kelp. Artificial kelp attached to a 3.6 kg vinyl-coated kettle bell and on the artificial kelp, one 11.4 cm and two 15.2 cm jollyballs with drilled 5.1 cm holes were attached using paracord. The problem-solving goal was to pull fish out of the jollyballs suspended on the artificial kelp in the water column. (**B**) Feed box. Blueprint designed by UC Davis Biomedical Engineering department that used 63.5 × 53.34 cm PVC and starboard plastic panels with six hinged flaps. The problem-solving goal was to lift the hinged flaps to retrieve fish inside the compartments at the bottom of the pool. (**C**) Fish cocktail. A 7.5 L plastic water jug with 5.1 cm holes drilled throughout and artificial kelp strung through the water jug and secured with a 3.6 kg vinyl-coated dive weight at the bottom and two 12.7 × 7.6 cm plastic pool floats at the top. The problem-solving goal was to maneuver the jug and pull the fish from the jug that was suspended in the water column. (**D**) Honeycomb. A 38.1 × 50.8 × 15.2 cm medium-duty plastic box with 5.1 cm holes in a grid formation on the front and sides of box from Otto Environmental. The problem-solving goal was to maneuver the box and pull fish from the box floating at the surface. (**E**) Kelp forest. A 121.9 × 60.9 cm PVC frame with a cross section that secured two 25.4 cm jollyballs with drilled 5.1 cm holes secured by paracord and artificial kelp dangled from each side of the frame. The problem-solving goal was to retrieve fish from the jollyballs as the frame floated at the surface and the balls were suspended just underneath the water. (**F**). PVC reef. A 33.0 × 27.9 × 48.2 cm milkcrate secured with an 8-lb vinyl-coated dive weight on the inside and a capped PVC pipe with three 5.1 cm holes attached to the outside with artificial kelp secured on each side of the crate. The problem-solving goal was to retrieve fish that was inside the PVC pipe that was weighted at the bottom of the pool.

We built twelve enrichment devices with a focus on ecological relevance, durability, animal safety, and easy disinfection and cleaning. Six structural enrichment devices (Figure 2) provided environmental complexity, and six cognitive enrichment devices (Figure 3) provided environmental complexity but additionally had a problem-solving goal of retrieving 0.2 kg of whole, thawed herring. For these cognitive enrichment devices, the herring was placed inside the device (specifics described in Figure 3) at the start of the enrichment session, allowing seals to interact with the device to retrieve the food reward. Structural enrichment devices did not contain food.

#### 2.2.3. Feeding Sessions

Feeding sessions took place three times per day (8:00, 13:00, 21:00). Staff assigned seals to enclosures with conspecifics of similar foraging skill, separating younger seals who required tube feedings, and free-feeding seals that independently foraged for thawed fish delivered into the pool. Aside from feedings, animal care crews entered the enclosures throughout the day to rinse surfaces, siphon the pools, disinfect enclosures, and provide enrichment. As anticipatory behavior is expressed prior to predictable, rewarding events, we wanted to provide an unequivocal cue that the seals could associate with feeds and not other husbandry interactions to elicit anticipatory behavior.

Due to the variability of feeding duration with tube and hand-feeds, we measured anticipatory behavior once harbor seals were free-feeding. To capture anticipation towards feeding sessions, we created an unequivocal cue regarding the arrival of the feed by attaching an extended garden hose nozzle outside of the pen gate that sprayed water over each pool. Prior to each feed, animal care personnel turned on the hose to allow a continuous water spray for one minute over the pool to signal that feeding time was coming. After one-minute, crews turned off the shower cue, entered the pen, dispensed the frozen/thawed herring pre-weighed based on the dietary needs of each seal into the pool using a scatter-feeding method that allowed seals to feed independently as staff exited the pen. We used Axis P3245-LVE cameras to record feeding sessions that we downloaded remotely for later behavioral analysis.

### 2.3. Data Collection

#### 2.3.1. Video Data Sampling

We collected video data four times per day for each of the five pens from 2 May 2021 to 6 July 2021 and from each of the six pens from 12 March 2022 to 11 July 2022. Due to technical difficulties, we lost 25 days of video data in 2021 and 44 days of video data in the 2022 season. However, we recorded 586 × 30 min enrichment videos and 203 × 30 min feeding videos across both seasons.

Since videos were of the pens and recorded multiple subjects, we required all seals to be individually identified. To identify each focal seal for behavior coding, a team of fourteen research assistants met seal identification reliability, whereby they used a combination of individualized spot patterns, colored hat tags, and flipper tag location to ensure the correct identification for each of the fourteen seals in all the videos. If a seal was not reliably identifiable in a video, we did not include that session in the seal’s dataset. After we established all seal identification, we reliably identified seals in 465 enrichment sessions and 201 feeding sessions.

#### 2.3.2. Enrichment Video Sampling

To accommodate that the seals had some choice and control over when and how long they interacted with enrichment during session, we randomly sampled video segments within each 30 min session for behavior coding. For each enrichment video, we identified which seals were present in the enrichment session. Then for each seal, we randomly sampled a 5 min segment within the 30 min video. We randomized the video samples by assigning 10 s interval start times a number beginning 1 min after the 30 min video started (e.g., 1:00 = 1, 1:10 = 2, 1:20 = 3, etc.) up to the 25 min start period (25:00 = 145). We then assigned a random number 1–145 to each enrichment session to obtain our randomized start times and then added 5 min to the start time to obtain each video sample (e.g., 24:30 start time +5 min = 24:30 start time to 29:30 end time to be coded). This video sample was then used to code all seals’ behaviors for each session.

If any human involvement occurred during that video sample (i.e., crew beginning to clean the pen, veterinary staff coming in to check on a seal), then we subtracted 7 min from the randomized start time and that would become the new start time in which we would begin coding (e.g., 24:30 start time—7 min = 17:30 start time to 22:30 end time).

#### 2.3.3. Feeding Video Sampling

We recorded feeding sessions based on the shower cue implementation, so we did not randomly sample videos segments but clipped 7 min prior to fish being delivered into the water and 3 min after crews delivered the fish into the water to be coded. While we originally wanted to use the shower cue as the time point to evenly divide the feeding videos to pre- and post-cue, there was variability in how the individual crews used the cue daily and how quickly crews delivered the fish after the cue turned off. To capture an accurate measure of anticipatory behavior, we sampled the videos pre-reward (7 min before the crews delivered the first fish to record any crew variation in the implementation of the cue), and post-reward (3 min after the crews delivered the first fish). This allowed us to compare behavior during the appetitive phase and after consumption.

#### 2.3.4. Behavioral Coding

We developed an ethogram (Table 1) to measure the harbor seals’ behavior during the enrichment and feeding sessions and used BORIS behavior coding software (version 7.9.7) to code the behaviors [40]. Seven research assistants passed behavior coding training for 13 behaviors using BORIS. We calculated interrater reliability from a series of eighteen video segments with the first author used as the exemplar. We evaluated all behaviors using two-way mixed intraclass correlation coefficients (ICC-3), collectively coding a video series at the beginning of data collection and another series half-way through data collection. For all measures, across both timepoints, coefficients remained in the excellent range (b = 0.85 to 0.96) for all assistants. Additionally, the first author visually inspected BORIS behavior event plots for alignment of behavioral events.

### 2.4. Data Analysis

#### 2.4.1. Measure: Enrichment Interaction Score

To measure each seal’s overall interaction with enrichment of either type (structural or cognitive), we summarized the total duration of “Device Interaction” behaviors and “Alert-Device” behaviors and divided the duration by the total video-clip time for each enrichment session. Clip time was calculated by taking the 5 min (300 s) video clip and subtracting any “Out of View” durations. Thus, the final score was the proportion of time spent interacting with enrichment for each session. We then used the arc-sine square root transformation to stabilize variance and normalize distribution of proportional enrichment interaction score data as this transformation is designed to handle values between 0 and 1.

#### 2.4.2. Measure: Enrichment Doses

During both seasons, we were limited by the available pre-release enclosure space. In 2021, we could only place seals into one pre-release enclosure for most of the season, where we distributed both structural and cognitive enrichment devices. In 2022, pool filtration issues resulted in our access to only one pre-release enclosure for certain time periods within the season. Once seals from designated structural or cognitive enrichment pools transitioned to the pre-release pen, we randomly assigned one of the total twelve enrichment devices (six structural, six cognitive) for each enrichment session to ensure that enrichment exposure remained variable but balanced across individuals. As a result, we exposed most seals to both cognitive and structural enrichment, meaning we could not use enrichment condition as a categorical variable.

**Figure 4:**
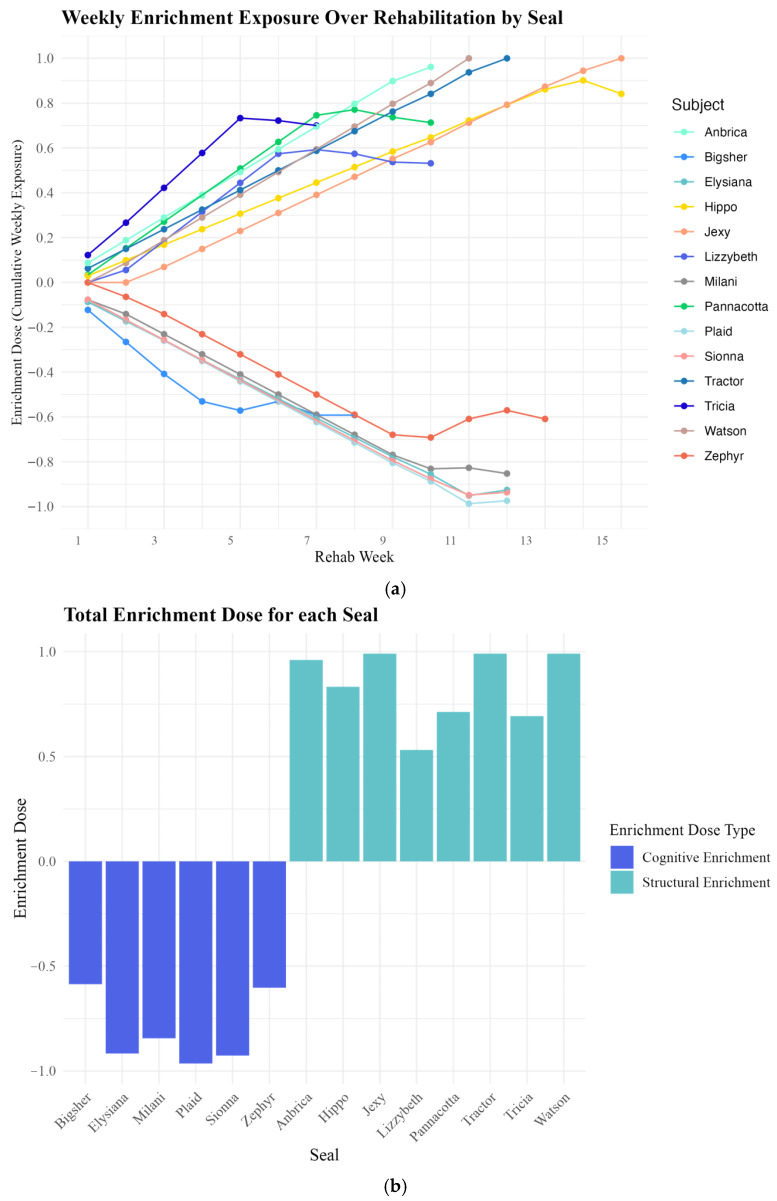
(**a**,**b**). Seal Enrichment Doses: (**a**) Weekly enrichment doses across rehabilitation. Weekly cumulative enrichment doses for individual seals across the rehabilitation period (X-axis: rehabilitation week, Y-axis: enrichment dose). Each line represents a seal’s enrichment dose trajectory, with positive values indicating structural enrichment and negative values indicating cognitive enrichment. The increasing absolute value of enrichment doses over time reflect the growing exposure to one type of enrichment as seals progressed through rehabilitation. Curves in the lines indicate shifts in enrichment type, with most changes occurring in later weeks, coinciding with seals’ transition to pre-release enclosures where exposure to different enrichment types was more likely. (**b**) Cumulative seal enrichment doses. Cumulative enrichment doses for individual seals (X-axis) are shown, with enrichment dose values (Y-axis) ranging from −1 to +1. Bars below zero (purple) represent seals with predominantly cognitive enrichment doses (average = −0.81, SD = 0.17, *n* = 6), while bars above zero (aqua) represent seals with predominantly structural enrichment doses (average = 0.84, SD = 0.18, *n* = 8). The distribution of doses indicates that seals fell into two distinct enrichment categories, highlighting the continuous nature of the enrichment dose scale and the clustering of average doses by type.

Instead, we created a cumulative enrichment dose profile (Equation (1)) to capture how much exposure to each type of enrichment each seal received during rehabilitation. We created an enrichment dose using each seal’s number of structural enrichment exposures (*EnrichmentExposure_structural_*), number of cognitive enrichment exposures (*Enrichment Exposure_cognitive_*), and days the individual was in the study (*Study Days*). The numerator took the total cognitive enrichment exposure and divided by total days in the study and subtracted that number from the total structural enrichment exposure divided by total days in the study. The denominator took the maximum enrichment exposure of both types divided by the maximum days in the study for all subjects. We also scaled this enrichment dose profile (Equation (2)) to capture the enrichment exposure as the weeks progressed to monitor when exposures to differing enrichment types occurred (Figure 4a).

(1) $$Enrichment\;{Dose}_{seal}=\frac{{\left(\frac{Enrichment\;Exposure_{structural}}{Study\;Days}\right)}_{total}-{\left(\frac{Enrichment\;Exposure_{cognitive}}{Study\;Days}\right)}_{total}}{\begin{array}{c} {\left(\frac{Enrichment\;Exposure_{structural}+Enrichment\;Exposure_{cognitive}}{Study\;Days}\right)}_{max\;total} \end{array}}$$

Equation (1): Cumulative enrichment dose formula across rehabilitation.

(2) $$Enrichment\;{Dose}_{week}=\frac{{\left(\frac{Enrichment\;Exposure_{structural}}{Study\;Days}\right)}_{week}-{\left(\frac{Enrichment\;Exposure_{cognitive}}{Study\;Days}\right)}_{week}}{\begin{array}{c} {\left(\frac{Enrichment\;Exposure_{structural}+Enrichment\;Exposure_{cognitive}}{Study\;Days}\right)}_{max\;week} \end{array}}$$

Equation (2): Weekly enrichment dose formula across each week in rehabilitation.

These formulas turned enrichment exposure into a continuous variable from −1 to +1, whereby seals that were in rehabilitation for longer time periods and exposed to predominantly cognitive enrichment had a more negative number and seals that were in rehabilitation for longer time periods and exposed to predominantly structural enrichment had a higher positive number. A − 1 dose indicated the longest exposure to only cognitive enrichment and a + 1 dose indicated the longest exposure to only structural enrichment (Figure 4b).

As most exposures to the different enrichment types occurred once seals were in the pre-release pens, the data indicates two clear clusters exposed to predominantly structural and predominantly cognitive enrichment types (Figure 4b). While we treat the enrichment dose measures as a continuous variable, we referred to any doses less than zero as “cognitive doses” and any doses more than zero as “structural doses.”

#### 2.4.3. Measure: Anticipatory Behavior Rates

To compare the overall intensity of anticipation prior to feeding sessions, we measured anticipatory behavior as two distinct variables. Anticipatory behavior count, which reflects the frequency of transitions between behaviors, and anticipatory behavior duration which represents the cumulative time spent in distinct anticipatory behavior bouts. Below we follow the quantitative methods outlined by Podturkin, Krebs, and Watters (2023) to create these anticipatory behavior rates [28].

The first step was to establish pre-reward and post-reward time clips for each feeding session, with pre-reward as 3 min prior to the first fish delivery and post-reward as the 3 min after first fish delivery. We then summarized all the counts and durations of each behavior that occurred during each pre-reward and post-reward period. We checked these data for normality using Kolmogorov–Smirnov tests but data were not normally distributed, so we used nonparametric tests for our analyses.

The next step was to determine if any feeding sessions should not be included in the dataset. During behavioral coding, the data collection team noted any abnormal sessions such as use of enrichment during a feeding session, crew variation (e.g., repeated entry and exit, cues turning on and off, feeding delay after the cue was turned off), and any session where the cue was not used during a feeding session. Out of the 201 feeding sessions coded, 2 sessions used enrichment during the feed, 46 sessions were labeled as having crew variability, and 14 sessions did not use the shower cue. We automatically dropped the 2 sessions that used enrichment during the feed and then we used nonparametric Mann–Whitney U unpaired *t*-tests to assess if crew variability or the lack of cue influenced the seals’ behavior and needed to be excluded from the data. When we compared whether crew variability did or did not occur during a feeding session, we found no significant difference in the behavior expression for both the behavior counts (*W* = 3075, *p* = 0.35) and durations (*W* = 3177, *p* = 0.22). When we tested if the lack of a cue influenced behavior expression during feeding sessions, we found a significant difference for both count (*W* = 876, *p* = 0.05) and duration (*W* = 3075, *p* = 0.04) behaviors when the cue was not used versus when the cue was used. Thus, we excluded the 14 sessions where the cue was not used and had a total of 185 feeding sessions in our dataset.

Once we removed any sessions without a cue, we ran Wilcoxon-rank paired *t*-tests to compare the expression of behaviors during the pre-reward versus post-reward period to establish which behaviors were anticipatory. Due to the need to run multiple *t*-tests for both the count and duration of behaviors and their modifiers in our ethogram, we employed a Bonferroni correction to adjust significance levels for the count (α/17) and duration behaviors (α/10) to ensure stringent control of the family wise error rate. The Wilcoxon-rank paired *t*-tests with the Bonferroni correction revealed significant differences in six of the count behavior-modifier combinations and five of the duration behavior-modifier combinations, highlighted in Table 2.

The significant differences in the count behavior-modifier combinations indicated that “Alert-Beyond Pen”, “Alert-Door”, “Alert-Seal”, “Crowd-Seal”, “Galumph”, “Haul-Out”, “Spyhop”, and “Water Entry” occurred during the pre-reward period at greater frequency than the post-reward period. The significant differences in the duration behavior-modifier combinations indicated that “Alert-Beyond Pen”, “Alert-Door”, “Alert-Human”, “Alert-Seal”, and “Galumph” occurred during the pre-reward period for longer durations than the post-reward period. Because anticipatory behavior occurs at a greater frequency or duration prior to the reward, we considered any behavior with higher rates in the pre-reward period as anticipatory. Thus, while “Prey Interaction” and “Prey Feed” were also significantly different pre- and post-reward, the total counts and durations indicated the behaviors were not anticipatory because there were higher rates in the post-reward period.

Finally, to assess the intensity of the seals’ anticipatory behavior before feeding sessions, we quantified two measures, “anticipatory behavior count rate” and “average anticipatory behavior duration”. We calculated count rate by summing all occurrences of established anticipatory behaviors during each feeding session and dividing by the pre-reward period (180 s). Similarly, we determined average duration by dividing the cumulative duration of anticipatory behaviors by the same pre-reward period. We then calculated weekly rates for each seal by summing anticipatory behaviors across sessions for the week and dividing by the total pre-reward time for that week.

### 2.5. Statistical Analyses

To analyze the effects of enrichment on anticipatory behavior rates while accounting for potential random effects, we used linear mixed models (LMM). This approach allows us to model both fixed effects (e.g., enrichment interaction or enrichment dose) and random effects (e.g., individual seal or weeks in rehabilitation). LMM also accounts for the non-independence of hierarchical data and unbalanced data, resulting in robust models that can be generalized beyond the specific study sample. We used RStudio (version 2023.09.1+494) to run our analyses with the “lmerTest” package (version 3.1.3).

For both hypotheses, we visually inspected our data using “dotchart” function in R and no extreme values or outliers were identified in any variable that may skew results. We tested collinearity among the predictor variables and found VIF values to be low (<2), indicating no multicollinearity concerns. Finally, we checked model fits for normality and homogeneity using QQ plots and residual diagnostics (“hist”, “plot”, and “resid” functions in R) with all assumptions met unless otherwise stated.

**Hypothesis** **1.** *Predictive hypothesis.*

To evaluate whether overall enrichment interaction (both structural and cognitive) predicted anticipatory behavior rates (H_1_), we fit two separate LMMs for “anticipatory behavior count rate” and “average anticipatory behavior duration” as the outcome variables. For both models, “enrichment interaction” was the continuous predictor variable and “seal” was the random effect for both models to account for repeated measures.

**Hypothesis** **2.** *Model selection.*

To identify the best-fitting model for predicting the effects of enrichment interaction and enrichment dose on anticipatory behavior rates (H_2_), we compared several candidate models using a hypothesis-driven approach and selected the final model based on a balance between goodness of fit, AIC values, and theoretical considerations. We fitted two models with the “anticipatory behavior count rate” and “average anticipatory behavior duration” as the outcome variables, with “enrichment interaction,” “enrichment dose,” and their interaction as fixed effects. Initially, we included “rehab week” and “seal” as random effects to account for within-subject variability and temporal structure of the rehabilitation period. The “rehab week” variable represented the number of weeks a seal had been in rehabilitation at the time of measurement (not the total duration of care); however, “rehab week” contributed negligible variability and thus we removed it from our models.

For both “anticipatory behavior count rate” and “average anticipatory behavior duration” models, we included “enrichment interaction” and “enrichment dose” as the fixed effects and “seal” and the random effect in our final models.

## 3. Results

We aimed to test the efficacy of using anticipatory behavior as a welfare metric in a wildlife rehabilitation setting using either structural or cognitive enrichment to modulate the welfare of rehabilitating harbor seal pups. We provided seals with daily structural or cognitive enrichment. We first established how anticipatory behavior manifested in the rehabilitating seals prior to scheduled feedings. We then measured the frequency and durations of these established behaviors to identify if enrichment types modulate the seals’ anticipatory behavior. Overall, results indicate that while the amount of enrichment interaction does not influence anticipatory behavior rates, the type of enrichment a seal is exposed to during rehabilitation affects average anticipatory behavior duration; seals who were exposed to predominantly cognitive enrichment exhibited lower anticipatory behavior than seals exposed to predominantly structural enrichment.

### 3.1. Enrichment Interaction Modulates Anticipatory Behavior (H_1_)

For our first hypothesis, we predicted that higher amounts of enrichment interaction (regardless of enrichment type) will predict lower anticipatory behavior rates. To test this hypothesis, we averaged “anticipatory behavior count rates” and “average anticipatory behavior duration” from each session by week and modeled them as a response variable in a linear mixed model. We also averaged “enrichment interaction” from each session by week. We first tested “anticipatory behavior count rate” as the outcome variable, with “enrichment interaction” as the fixed effect and “seal” was the random effect. Enrichment interaction was not a statistically significant predictor of “anticipatory behavior count rate” (*t* = −0.949, *p* = 0.35).

We then tested “average anticipatory behavior duration” as the outcome variable, with “enrichment interaction” as the fixed effect and “seal” as the random effect. We found that enrichment interaction was not a statistically significant (*t* = 0.66, *p* = 0.51) predictor of “average anticipatory behavior duration”.

**Figure 5:**
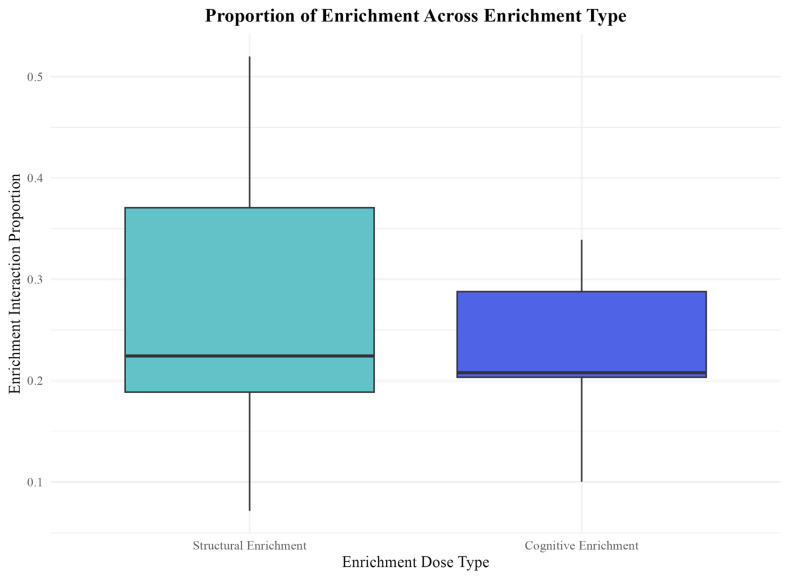
Proportion of enrichment interaction across enrichment types. Enrichment interaction was not significantly different between enrichment types. The y-axis represents raw proportion of time spent interacting with enrichment, while the arcsine square-root transformation of these values was used for statistical analyses. The boxplot displays the median proportion of enrichment interaction for each enrichment type, with the interquartile range represented by the box and whiskers indicating data variability. Seals interacted with structural enrichment for a median proportion of 0.228 and seals interacted with cognitive enrichment for a median proportion of 0.207 during recorded sessions.

Although “enrichment interaction” was not a significant predictor of either anticipatory behavior measure, we further examined how often seals interacted with the different types of enrichment to explore potential underlying factors. Interestingly, we found the proportions of interaction with cognitive and structural enrichment were quite similar. On average, seals interacted with structural enrichment 28% of the coded periods and 23% of the time with cognitive enrichment (Figure 5). This similarity in interaction rates suggests that the type of enrichment, rather than the overall level of interaction, may be the driving factor influencing anticipatory behavior.

### 3.2. Enrichment Dose Modulates Anticipatory Behavior (H_2_)

**Figure 6:**
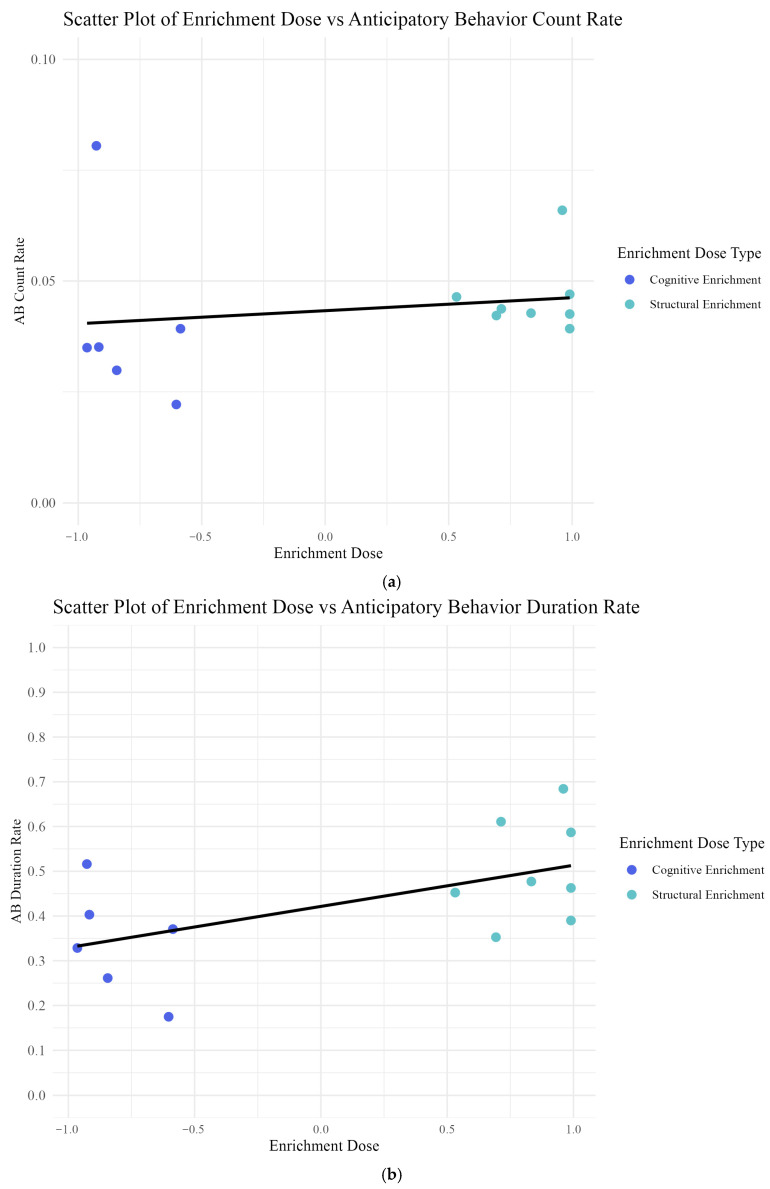
(**a**,**b**) Relationship between enrichment dose and anticipatory behavior measures. (**a**) Relationship between anticipatory behavior count rate and enrichment dose. As cognitive enrichment doses occur below zero and structural enrichment doses occur above zero, datapoints are color-coded by enrichment type: cognitive (purple) and structural (aqua). The fit line indicates no significant relationship. One purple data point at 0.08 represents Sionna, who had a notably high count rate, regardless of enrichment dose. This is likely because her anticipatory behavior expression manifested as a frequent spyhopping behavior prior to feeding sessions, which highlights individual variability. (**b**) Relationship between average anticipatory behavior duration and enrichment dose. The fit line represents trends identified in our linear mixed model analysis that seals exposed to predominantly cognitive enrichment (lower, negative doses), exhibited shorter average durations of anticipatory behavior, while seals exposed to predominantly structural enrichment (higher, positive doses) showed longer durations of anticipatory behavior. This pattern suggests a positive relationship between enrichment dose and anticipatory behavior duration, with seals’ enrichment exposure type influencing their anticipatory behavior intensity.

For our second hypothesis, we predicted that exposure to a predominantly cognitive enrichment dose would result in decreased rates of anticipatory behavior compared to a predominantly structural enrichment dose exposure. We tested this using the same methods as Hypothesis 1 and added the “enrichment dose” variable into the model. We found that neither “enrichment interaction” (*t* = −0.937, *p* = 0.355) nor “enrichment dose” (*t* = 0.435, *p* = 0.670) were significant predictors of “anticipatory behavior count rate” (Figure 6a).

When we tested whether “enrichment interaction” and “enrichment dose” predicted “average anticipatory behavior duration”, we found that, consistent with H_1_, “enrichment interaction (*t* = 0.629, *p* = 0.533) was not a significant predictor of “average anticipatory behavior duration.” While “enrichment dose” was not statistically significant (*t* = 2.049, *p* = 0.057) we consider it a biologically relevant predictor for “average anticipatory behavior duration,” given the small number of individuals tested.

Although “enrichment interaction” was not statistically significant in predicting either “anticipatory behavior count rate” or “average anticipatory behavior duration,” we retained it in the final models due to its biological relevance and its impact on model fit. Specifically, removing “enrichment interaction” resulted in a marginally lower AIC for the “duration rate” model but increased residual skewness and reduced the significance of “enrichment dose” (from *p* = 0.057 to *p* = 0.15). Retaining the variable improved interpretability and ensured a biologically meaningful model. In conclusion, while we found no effect of enrichment interaction on anticipatory behavior (either count rate or average duration), we did find a trending effect of enrichment dose on average anticipatory behavior duration (Figure 6b).

## 4. Discussion

In this study, we tested the ability of using anticipatory behavior as an effective metric of welfare for seals in a rehabilitation setting. To test this, we exposed seals to daily structural and cognitive enrichment sessions, and prior to scheduled feeding sessions, we observed their anticipatory behavior throughout their rehabilitation. Our results first established which behaviors were anticipatory as expressed by harbor seal pups and then quantified them into one anticipatory behavior measure. Using this measure, we found a trend (*p* = 0.057) showing that the enrichment dose (i.e., the type and amount of enrichment exposure) modulated anticipatory behavior, with exposure to more cognitive enrichment reducing the duration of anticipatory behavior more than exposure to structural enrichment. However, perhaps because seals used both types of enrichment for nearly equal amounts of time, we did not find evidence that the amount of enrichment interaction influences anticipatory behavior rates.

While our sample size was necessarily limited by the nature of wildlife rehabilitation, this study provides valuable insights into potential psychological welfare assessments and insights into enrichment effects in this unique population. Unlike traditional research settings, we cannot select our subjects; rehabilitation research must work with the animals admitted into care and within constraints of this temporary environment. To control extraneous factors, we focused on a standardized subset of animals with the most similar health circumstances, ensuring the most robust and interpretable findings. Our results suggesting that cognitive enrichment reduces anticipatory behavior more than structural enrichment adds to the growing evidence that using anticipatory behavior is an effective welfare assessment tool and enrichment, more specifically that cognitive enrichment, as a husbandry tool improves the welfare of seals in a rehabilitation setting.

### 4.1. Measuring Welfare: Anticipatory Behavior

Anticipatory behavior, while becoming a more common welfare metric in other managed care settings, is still a novel measurement for the welfare of animals in wildlife rehabilitation; to our knowledge only one other study measured anticipatory behavior in this setting (West Indian manatee, *Trichechus manatus manatus*) [34]. Feeding schedules are an essential part of wildlife rehabilitation husbandry and inevitable environmental cues (i.e., sounds or increased human presence) that are coupled with subsequent feedings make the anticipation of feeding events an opportune period to examine the welfare of harbor seal pups. Once seals began free-feeding, we paired a 1 min shower cue over the pool immediately prior to each feeding session to provide a salient cue for the upcoming feeding event and recorded their behaviors pre- and post- reward (when fish was given). Our data are consistent with literature that establishes anticipatory behavior as a suite of behaviors that are related to the appetitive, information-gathering part of a behavioral sequence with the ultimate goal of resource acquisition [27,43]. Some behaviors that emerged as anticipatory also suggest that the behaviors expressed depend on space, context, and time [26].

The behaviors “Alert-Beyond Pen” and “Alert-Door” were all very prevalent in the pre-reward period compared to the post-reward period with “Alert-Beyond Pen” occurring four times more often (count) and almost six times longer (duration) in the pre-reward period than post-reward and “Alert-Door” occurring five times more often and over nine times longer in the pre-reward period (Table 2). The seals’ behavior expression is consistent with investigatory behaviors that would be seen in the appetitive phase as there is only one door where crews enter to feed and general activity just beyond the pen increases prior to feeds. To reduce habituation, all pen doors and fences are covered with dense mesh to limit visual sensory cues. Animal care personnel also limit the time they are active around the rehabilitation pens other than necessary husbandry events such as feeds, pen cleaning, and veterinary assessments. However, reliable cues that consistently occur prior to an event, whether intentionally or inadvertently, are still cues that provide information for which an animal can act upon [44]. Environmental cues associated with feed event preparation (e.g., increased human presence, food bucket sounds, slickers and boots being washed before entry) outside the pens are apparent, so it is not surprising to observe increased focus from the seals towards locations where the fish reward is coming from. “Spyhop” behavior was double the frequency in the pre-reward period, and similar to dolphin anticipatory behavior, spyhopping can be considered an investigatory behavior [39,45].

While there was increased focus towards the spaces in the seals’ enclosure where rewards emerged, increased on-land locomotion occurred in the pre-reward period as well. “Galumph” was expressed nine times more often and almost eight times longer in the pre-reward period than the post-reward, despite fish always being delivered into the water. The point-event behavior “Water Entry” occurred six times more often in the pre-reward period than post-reward period (Table 2). These behavior patterns also provide insight into how space and context influence anticipatory behavior. We did not expose harbor seals to the shower cue until they were feeding independently (also known as free-feeding). When learning how to free-feed, seals are brought on deck or in shallow-water pools to be hand-fed fish; however, as soon as seals free-feed, whole, thawed herring are distributed into the pool for seals to “catch” and eat themselves. The increased galumphing and water entry is an indication that seals, if on deck resting, would make their way into the pools where they know the fish reward will be.

While not significant, it is interesting to point out the increased “Haul-Out” and “Crowd-Seal” behaviors that occurred at double the frequency in the pre-reward period (Table 2) These behaviors, in addition to “Alert-Seal” behaviors occurring over 5 times longer in the pre-reward period, we believe aim to collect information about the environment and are artifacts of the rehabilitation space. Harbor seals were housed in two types of enclosures during their rehabilitation, in-ground hospital pools, and above-ground pre-release pools. In the in-ground pools, frequent haul-outs would be observed prior to the feed as the seals would go towards the door (where the reward first enters the enclosure via crews) and transition into the water (where fish was dispersed). In the above-ground pools, frequent haul-outs and water entries would occur because there was only one pool entry and exit point which was at the top of the ramp and the highest vantage point to see the door (where the reward first enters) while also being closest to the entry of the pool (where the fish was dispersed). As space within these vantage points to obtain information about the upcoming feeding session were limited, “Alert-Seal” and “Crowd-Seal” are artifacts of the rehabilitation space.

Establishing and quantifying seals’ anticipatory behavior provides rehabilitators with a valuable tool for assessing animal welfare, offering advantages over relying solely on stereotypical behavior or abnormal repetitive behaviors (ARBs). Unlike stereotypical behaviors, which often emerge as a coping mechanism to prolonged stress, frustration, or boredom, anticipatory behaviors can provide a more immediate and dynamic welfare assessment. In rehabilitation the temporary nature of care may mean animals do not exhibit stereotypical behaviors as frequently, and if they do occur they must be opportunistically observed and only indicate negative welfare [16]. Alternatively, anticipatory behaviors can be elicited methodically through environmental cues, providing a proactive “temperature check” on an animal’s welfare state. Because it reflects reward sensitivity, anticipatory behavior acts as a form of “self-report,” reflecting the seals’ own perceptions of rewarding opportunities in their environment that captures welfare on a continuum rather than a one-dimensional scale. Understanding how seals perceive their surroundings in real time can help rehabilitators make welfare-informed adjustments to modulate anticipatory behavior. As demonstrated in this study, such behavioral responses can be used to evaluate the impact of varying environmental stimuli, like enrichment types, on their psychological welfare, offering a non-invasive, practical approach to monitoring and improving welfare in rehabilitation.

### 4.2. Improving Welfare: Enrichment

We found that the physical interaction with enrichment is not correlated with reduced anticipatory behavior rates (H_1_) but there is a tendency for enrichment dose (predominantly cognitive) to predict reduced “average anticipatory behavior duration” (H_2_). Anticipatory behavior precedes access to resources or events that are rewarding for the animal, with higher intensity expressed in reward-deprived environments and lower intensity expressed by animals in environments with more rewarding opportunities [27]. Environments with more rewarding opportunities can include enriched environments. Rats (*Rattus norvegicus*) in standard laboratory housing responded to a sucrose reward with higher anticipatory behavior than rats housed in enriched environments [46]. Another study found that rats in standard-housed environments expressed more anticipatory behavior towards a food reward than rats housed in semi-naturalistic conditions [47]. Cows (*Bos taurus*) housed in standard environments expressed more anticipatory behavior in response to access to a reward pen than animals housed in enriched environments [32]. Our results dive deeper into this concept by asking the seals if certain types of enrichment are more rewarding.

While enrichment dose was a continuous variable, two groups emerged as either having predominant exposure to cognitive enrichment or predominant exposure to structural enrichment which have similar absolute values, indicating that the exposure amount for each respective enrichment are quite similar. Although we exposed seals to predominantly one type of enrichment, the seals’ proportion of time interacting with the enrichment was similar for cognitive enrichment dose (*M* = 0.228, *SD* = 0.086) and structural enrichment dose (*M* = 0.276, *SD* = 0.150). Yet exposure to predominantly cognitive enrichment showed a trend towards reducing average anticipatory behavior duration (*p* = 0.057). This suggests that cognitive enrichment may influence behavioral responses more effectively than structural enrichment, though the effect does not meet conventional significance thresholds in this study. Although the effect of enrichment does was not statistically significant, we consider it biologically meaningful given the conservative nature of our study design. We compared two types of enrichment rather than contrasting enrichment treatment group and no-enrichment control group, so we are not surprised that observable differences are subtle. The fact that exposure to predominantly cognitive enrichment still showed a reduction in anticipatory behavior duration suggests that cognitive enrichment may have a stronger influence welfare-related behaviors than structural enrichment.

The effectiveness framework may provide insight into this pattern as it proposes that animals are motivated to be successfully engaged with their environment [48]. Being successfully engaged with the environment looks like effectively gathering information (truth effectiveness), gaining rewards (value effectiveness), and managing themselves within their environment (control effectiveness) [48]. While seals may experience information effectiveness by gathering information from interacting with both structural and cognitive enrichment, solving problems and actively working to retrieve fish provides additional rewarding experiences beyond only gathering information. In addition to information effectiveness, cognitive enrichment provides value effectiveness through collective hedonic experiences of decision value, remembered value, and experience value where seals use their choice to interact with the device, their learned experiences when interacting with the device, and the physical experience of interacting and solving a problem [48]. Cognitive enrichment also provides the experience of control effectiveness; by collecting information and retrieving rewards based on that information, seals can then use this to exert more control over their environment.

Like the effectiveness framework, the ethological needs model shows that animals are internally motivated to explore and learn as part of the goal-directed behavioral sequence. If animals are unable to complete this sequence, from the anticipatory, goal-seeking phase (appetitive) to the fulfillment of the goal (consummatory), their welfare may be compromised [49]. Behavior and evolutionary evidence also point to animals wanting and needing goal-directed, cognitive challenges [8,10]. Contrafreeloading is a behavior phenomenon seen in managed care settings where animals seek to “work for food” when the same resources could be acquired “for free” [50]. Wild, free-ranging animals face daily challenges to find food, reproduce, establish shelter, and avoid predators. The ability for animals to build a toolbox of cognitive skills to interact adaptively with their environment is essential to survival.

Though seals’ enrichment interactions indicated that both structural and cognitive enrichment provided an equitable amount of stimulation during the enrichment sessions, our results suggest that cognitive enrichment offered a deeper, more rewarding engagement as evidenced by the reduced reward sensitivity prior to feeding sessions. These results support both the effectiveness framework and ethological needs model, emphasizing the value of goal-directed challenges promoting psychological welfare. By providing frequent opportunities for seals to actively solve problems and achieve success, cognitive enrichment not only meets their intrinsic motivations but prepares them for adaptive survival strategies.

## 5. Limitations

This study provides important findings about behavioral measures and management interventions to assess and improve animal welfare in rehabilitating harbor seal pups; however, several limitations should be considered. First, our sample size was small (*n* = 14) and we had no “true” control group. Without a control group that received no enrichment, our ability to detect the full range of enrichment effects on anticipatory behavior was limited. Therefore, this was a conservative test of the validity of anticipatory behavior as a measure of welfare because we expect a smaller effect of cognitive versus structural enrichment than we would between enrichment versus control. We were unable to include a control because Chudeau and colleagues (2019) already found that enrichment improves welfare over a control condition; therefore, it was unethical for us to purposefully withhold enrichment for this study [16]. It is critical to emphasize that the lack of statistical significance in this study should not be interpreted as enrichment having little to no impact on welfare, as the experiment was not designed to test for this effect. Instead, these results highlight the need for further research with larger sample sizes and perhaps alternative designs (e.g., ABA within-subject designs) to fully capture the relationship between types of enrichment and anticipatory behavior.

Additionally, while the one-minute shower cue duration ensured that all seals had an opportunity to perceive it, our observations suggest that this duration may have been too lengthy. Seals occasionally exhibited increased activity and anticipatory behavior at the beginning of the cue, activity decreased as the cue continued, then intensity would reemerge when the cue turned off, as if the cue ceasing was a secondary cue. Future studies and protocols might refine the cue duration to reduce ambiguity in responses.

It is also possible that not all pups responded exclusively to the intended shower cue, but rather to a signal string of reliable cues occurring alongside it, such as increased human activity or the behavior of penmates. Reliable cues may be inadvertent, and their salience can vary among individuals depending on context and position in the enclosure. While this likely does not alter the overall interpretation of our results, it represents the inherent complexity of working with perceptive animals in unique settings.

Furthermore, unlike other anticipatory behavior studies, a formal conditioning period to ensure that the cue was associated with the feed was not feasible for our study. The dynamic nature of the rehabilitation environment of patients continuously admitted throughout the season and beginning to independently feed at different times precluded a set conditioning period like traditional anticipatory behavior studies. Despite no conditioning period, we and the animal care personnel observed salient behavioral changes indicating that the seals effectively learned the cue-reward association within days of exposure, supporting the validity of our results. Finally, despite the mixed exposure to enrichment types, the use of the enrichment dose formula allowed us to quantify the cumulative intensity of the exposure to both types of enrichment. However, we recognize that since there was no control group, this measure may have made our test more conservative and limited our understanding of the full range of the relationship between enrichment and anticipatory behavior. This continuous variable preserved nuanced information that would have been lost with a simple categorical approach and enhanced the study’s ecological validity as it reflects real world rehabilitation practices where animals may receive various enrichment devices. Interestingly, most mixed exposures occurred in pre-release enclosures, raising the possibility that developmental factors or early experiences with problem-solving and reward acquisition may influence a seal’s overall welfare while in rehabilitation. Further studies could explore this hypothesis to better understand these potential dynamics.

## 6. Conclusions

Wildlife rehabilitation work strikes a delicate balance between the necessary human intervention for medical treatment and the potential impacts of these interventions on an animal’s welfare. Veterinary care is the critical first step in the rehabilitation process; however, greater emphasis on the reconditioning phase is essential for longer-term hospitalizations. Husbandry strategies that prepare animals physically, behaviorally, and psychologically are still evolving, and research aimed at improving them is in its infancy. Our study demonstrates that anticipatory behavior provides a viable, noninvasive measure of welfare based on how these wild seals perceive and respond to opportunities within the rehabilitation environment. Moreover, it highlights that cognitive enrichment has the potential to enhance animal welfare by addressing the psychological needs of young, rehabilitating seals.

Our findings underscore the importance of incorporating cognitive enrichment into regular husbandry practices. While cognitive enrichment is intrinsically rewarding, providing a variety of enrichment types is crucial. Structural enrichment allows animals to physically interact with their environment, fostering exploratory behaviors and physical agility that are critical for survival post-release. Without considering the functional significance for the animal, enrichment design will fail [12,51]. Effective enrichment requires more than introducing variability; it must deliver biologically relevant outcomes that balance sensory stimuli, challenge, and the opportunity for goal-directed interactions [10,52,53].

We also echo Clark’s (2023) call for animal caretakers to design and implement cognitive enrichment devices that prioritize manageable, biologically relevant challenges over extending foraging time or novelty alone [54]. Cognitive challenges encourage animals to recognize, learn, and adapt to environmental contingencies, and seek solutions to better understand and interact within their environment [10,55]. Such enrichment promotes learning through exploration and problem-solving, which can build the resilience and adaptability necessary for survival in dynamic, post-release environments [51,56].

In conclusion, this study highlights the promise of measuring anticipatory behavior in wildlife rehabilitation settings to provide insights into how animals perceive their environment and offers a practical approach for assessing welfare of wild animals in this unique setting. While this study design created a conservative test of anticipatory behavior as a welfare indicator, our results combined with prior research demonstrate enrichment’s ability to modulate welfare of rehabilitating seal pups [16]. Thus, integrating enrichment, particularly cognitive, into wildlife rehabilitation programs offers a path forward for more comprehensive welfare strategies. By utilizing anticipatory behavior, rehabilitators may have a real-time, noninvasive measure of psychological welfare that can inform management practices to address physical, behavioral, and psychological needs while in rehabilitation. This study highlights not only the potential of enrichment to bridge the rehabilitative and post-release phases but also the innovation of using anticipatory behavior as a noninvasive measure of psychological welfare. As we refine these tools and practices, we advance toward a future where wildlife rehabilitation not only restores health but fosters resilient, thriving animals better prepared for life in the wild.

## Figures and Tables

**Table 1:** Ethogram. We adapted the operational definitions for the following behaviors from other peer-reviewed ethograms: Spyhop [41], Swim, Device Interaction, and Pattern Swim [42], and Suckle [14].

Behavior	Modifiers	Behavior Type	Operational Definition
Crowd	Door, Human, Seal	Point	Seal is within 1 body-length of the modifier
Spyhop			Vertical or near-vertical elevation of body in the water with head up or backwards towards dorsal side and stationary movement
Haul-Out			Body (does not include back flippers) completely **out** of water
Water Entry			Body (does not include back flippers) completely **in** the water
Prey Feed	Underwater, At Surface		Thawed prey is consumed or swallowed
Alert	Beyond Pen, Device, Door, Human, Seal	State	Directed visual orientation towards modifier in an upright posture or lifted head
Device Interaction	Underwater, At Surface		Direct inspection, touch, and/or manipulation of device. Includes nosing with muzzle or vibrissae in an unpatterned fashion, mouthing, or touching device.
Prey Interaction	Underwater, At Surface		Manipulating prey. Includes contact with muzzle or vibrissae in an unpatterned fashion, mouthing, touching, or positioning prey
Suckle	Pen Structure, Seal		Attempt to latch onto modifier with muzzle or mouth actively pressed into modifier
Swim			Non-repetitive, locomotion in the water, varying in direction and speed; includes side-to-side motion of back flippers and/or motion of front flippers for propulsion forward or direction change with head above or below water
Galumph			Full body movement towards a different location on land; includes rolling, undulating, or maneuvering over objects
Out of View			Focal seal is out of view to identify behavior; includes out of camera view, obstructed by light reflection in the water and/or pen structures
Pattern Swim			Smooth locomotion in water at near constant speed in a repetitive pattern for > 2 complete revolutions of the pattern to complete “laps” of a part of the pool

**Table 2:** Established anticipatory behaviors identified through significant differences in the pre- and post- reward periods. Mean values and Wilcoxon rank-sum paired *t*-tests are reported with an asterisk (*) denoting significance after a Bonferroni correction. Behaviors classified as anticipatory occurred significantly more in the pre-reward period. Only behaviors with sufficient occurrences to allow for statistical comparison were included (e.g., Device Interaction was excluded as it did not occur during feeding sessions).

Behavior-Modifier	Behavior Metric	Pre-Reward	Post Reward	Wilcoxon Rank Paired *t*-Test	*p*-Value	Anticipatory Behavior?
		Mean (SD)				
Alert-Beyond Pen	Count	0.024 (0.009)	0.006 (0.005)	W = 105	0.001 *	yes
	Duration	0.249 (0.118)	0.043 (0.048)	W = 105	0.001 *	yes
Alert-Door	Count	0.010 (0.007)	0.002 (0.002)	W = 105	0.001 *	yes
	Duration	0.101 (0.087)	0.011 (0.024)	W = 105	0.001 *	yes
Alert-Human	Count	0.006 (0.003)	0.005 (0.003)	W = 61.5	0.084	no
	Duration	0.046 (0.028)	0.019 (0.015)	W = 100	0.003 *	yes
Alert-Seal	Count	0.003 (0.002)	0.0006 (0.0008)	W = 61	0.014	no
	Duration	0.016 (0.016)	0.003 (0.003)	W = 77	0.003 *	yes
Crowd-Door	Count	0.0004 (0.0008)	0.0001 (0.0003)	W = 15	0.059	no
	-	-	-	-	-	-
Crowd-Human	Count	0.0004 (0.0007)	0.0003 (0.0006)	W = 11.5	0.916	no
	-	-	-	-	-	-
Crowd-Seal	Count	0.004 (0.003)	0.002 (0.002)	W = 80.5	0.016	no
	-	-	-	-		
Galumph	Count	0.009 (0.010)	0.001 (0.002)	W = 105	0.001 *	yes
	Duration	0.031 (0.030)	0.004 (0.006)	W = 102	0.002 *	yes
Haul-Out	Count	0.002 (0.003)	0.001 (0.002)	W = 55	0.006	no
	-	-	-	-	-	-
Pattern Swim	Count	0.0005 (0.0008)	0.0001 (0.0003)	W = 20	0.058	no
	Duration	0.007 (0.012)	0.003 (0.013)	W = 19	0.093	no
Prey Interaction	Count	0.002 (0.002)	0.026 (0.008)	W = 0	0.001	yes
	Duration	0.011 (0.015)	0.144 (0.052)	W = 0	0.001	yes
Prey Feed	Count	0.0008 (0.001)	0.009 (0.003)	W = 0	0.001	yes
	-	-	-	-	-	-
Spyhop	Count	0.017 (0.006)	0.008 (0.004)	W = 105	0.001 *	yes
	-	-	-	-	-	-
Suckle-Pen Structure	Count	0.0001 (0.0002)	0.000 (0.000)	W = 6	0.174	no
	Duration	0.001 (0.002)	0.000 (0.000)	W = 6	0.181	no
Suckle-Seal	Count	0.0002 (0.0005)	0.0002 (0.0004)	W = 6	0.181	no
	Duration	0.003 (0.006)	0.0008 (0.002)	W = 10	0.1	no
Swim	Count	0.024 (0.011)	0.033 (0.010)	W = 13.5	0.016	no
	Duration	0.0414 (0.143)	0.526 (0.179)	W = 36	0.0315	no
Water Entry	Count	0.003 (0.002)	0.0005 (0.0006)	W = 91	0.002 *	yes
	-	-	-	-	-	-

## Data Availability

The raw data supporting the conclusions of this article will be made available by the authors on request.
